# Can Pancreatic Phlegmon be Diagnosed?

**DOI:** 10.1155/1990/68631

**Published:** 1990

**Authors:** Andrew L. Warshaw

**Affiliations:** Massachusetts General Hospital Wang Ambulatory Care Centre, Suite 336 Boston Massachusetts 02114 USA


### REFERENCES

1. Blumgart, L. H. (1988) Liver resection — liver and biliary tumors. In Surgery of the Liver and Biliary Tract, edited by L.H. Blumgart, vol. 2, pp. 1251–1280. Edinburgh: Churchill Livingstone
2. Bismuth, H. (1982) Surgical anatomy and anatomical surgery of the liver. *World Journal of Surgery*, **6**, 3–9
3. Huguet, C., Nordlinger, B., Bloch, P. and Conrad, J. (1978) Tolerance of human liver to prolonged normothermic ischaemia. *Archives of Surgery*, **113**, 1448–1451

### CAN PANCREATIC PHLEGMON BE DIAGNOSED?

### ABSTRACT

*Fan, S-t., Choi, T-k., Chan, F-l., Lai, E.C.S. and Wong, J. (1989) Pancreatic Phlegmon: What Is It? The American Journal of Surgery,* **157** 544–547

**In a retrospective study of 264 patients with acute pancreatitis, 22 were identified as having phlegmon by combined radiologic and clinical criteria. The radiologic criteria consisted of demonstration of abnormal lesion on computed tomography scan which was composed of masses of mixed density, free of extraluminal gas and lacking a well-defined wall. The clinical criteria was that the clinical course was free of sepsis. Half of the group thus identified had severe pancreatitis as defined as having three or more poor prognostic signs. Fever, leukocytosis, and serum amylase elevation persisted for a longer period than usual. Complication was infrequent but the lesion could persist for 3 to 4 months without producing symptoms. This is a relatively benign condition and surgery should be avoided**

### PAPER DISCUSSION

**KEYWORDS**: Pancreatitis, Pancreatic phlegmon

The term phlegmon seems to excite great interest among those attempting to describe acute pancreatitis and to understand its variegated clinical course. In large part that interest has been in trying to prove that the term is either bad or unuseful. There clearly has been no agreement on what the term means. Its original proponents used it primarily to denote a sterile process, with varying amounts of necrosis, but they also recognized that occult infection could be present or that infection could develop at a later time in the evolution of the disease.[1,2]

In the study by Fan *et al.*, the authors describe 22 patients who met their criteria of an abnormal inflammatory mass involving the pancreas and varying amounts of peripancreatic tissues, but without evidence of either pseudocyst or infection. Necrosis of the involved tissues was present in some of the cases and was associated with multi-system organ failure in four patients and death in three of those.

Secondary infection occurred in at least one patient, but perhaps would have been seen more frequently had antibiotics not been administered routinely. By definition and intent (and even with further selection in hindsight) these investigators eliminated patients with infected necrosis from their study group, thereby sharply skewing the possibility of finding infection in the series. In contrast Beger *et al.*[3] reported a prevalence of infection of nearly 40% in pancreatic and peripancreatic necrosis at the time of surgical debridement, findings which are very similar to those of Gerzof *et al.*[4] who studied pancreatic inflammatory masses with percutaneous needle aspiration (with no false-positives and no introduction of infection). Beger's group also showed that the clinical and hemodynamic manifestations of necrotizing pancreatitis are virtually indistinguishable whether or not infection is present.[5] What is clear from these studies is that the infection can be occult and that infected inflammatory masses may only be separated from sterile inflammation by direct bacteriological culture of the tissues, not by appearance alone. What is not yet known is how much necrotic tissue is tolerable or how to select those patients likely to heal without debridement and those for whom the risk of complication, including secondary infection, outweighs the risk of a surgical clean-out.

It has been my view that the element of *uncertainty* must be acknowledged by clinicians treating patients with acute pancreatitis.[6] It would be desirable (and soothing to our anxieties) to have black-and-white definitions and consequent absolute guidelines for treatment, but that is not what happens in the real world. The outcome of acute pancreatitis is unpredictable in a substantial number of patients. Patients with extreme fluid shifts of severe early pancreatitis, even to the point of shock, can recover without complication, while patients with a less impressive presentation can evolve to severe tissue injury, sepsis, and death. The prognostic scoring systems–Ranson, Glasgow, Bank, etc.–may be valid on a statistical basis for groups of patients, but all are unreliable predictors for individual patients. Sostre and his colleagues[7] made that point in their study of the phlegmon: the outcome could range from spontaneous resolution to complication, including later development of pseudocysts, abscesses, and death. It is agreed that most patients heal a phlegmon and do not need surgical intervention, but some do require that extra help. Even in the current study only 13 of the 22 patients had uncomplicated resolution of the process.

I continue to suggest that the major advantage of the term phlegmon should be its implication of uncertainty, of fragile truth which can change tomorrow, of the need to maintain vigilance lest the next act of the play go unobserved. Pancreatitis involves a temporal chain of events and injuries: early hemodynamic events, tissue necrosis, and later infection. These may be compressed into a few days or develop over a period of weeks. There is no evidence that a CT scan done on any single day (even with contrast enhancement)[8] can predict that entire course. Diagnosis of a phlegmon should imply only an interim impression and a provisional approach to therapy. It is the middle of the story, not its end.

Andrew L. Warshaw, M.D.
Professor of Surgery
Massachusetts General Hospital
Wang Ambulatory Care Centre, Suite 336
Boston, MASSACHUSETTS 02114
UNITED STATES OF AMERICA

### REFERENCES

1. Lawson, D.D., Daggett, W.M., Civetta, J.M., Corry, R.J. and Bartlett, M.K. (1970) Surgical treatment of acute necrotizing pancreatitis. *Annals of Surgery*, **172**, 605–617
2. Warshaw, A.L. (1974) Inflammatory masses following acute pancreatitis. Phlegmon, pseudocyst, and abscess. *Surgical Clinics of North America*, **54**, 922
3. Beger, H.G., Bittner, R., Block, S. *et al.* (1986) Bacterial contamination of pancreatitic necrosis. A prospective clinical study. *Gastroenterology*, **91**, 433
4. Gerzof, S.G., Banks, P.A., Robbins, A.H. *et al.* (1987) Early diagnosis of pancreatic infection by computed tomography-guided aspiration. *Gastroenterology*, **93**, 1315
5. Beger, H.G., Bittner, R., Buchler M. *et al.* (1986) Hemodynamic data pattern in patients with acute pancreatitis. *Gastroenterology*, **90**, 74
6. Warshaw, A.L. (1987) Lowering the level of uncertainty in late pancreatitis. *Gastroenterology*, **93**, 1434
7. Sostre, C.F., Flournoy, J.G., Bova, J.G. *et al.* (1985) Pancreatic phlegmon. Clinical features and course. *Digestive Diseases and Sciences*, **30**, 918
8. Bradley, E.L., Murphy, F., Ferguson C. (1989) Prediction of pancreatic necrosis by dynamic pancreatography. *Annals of Surgery*, **210**, 495

